# Comparative Genomic Analysis of *Campylobacter* Plasmids Identified in Food Isolates

**DOI:** 10.3390/microorganisms13010206

**Published:** 2025-01-18

**Authors:** Yiping He, Gretchen Elizabeth Dykes, Siddhartha Kanrar, Yanhong Liu, Nereus W. Gunther, Katrina L. Counihan, Joe Lee, Joseph A. Capobianco

**Affiliations:** 1 Characterization and Interventions for Foodborne Pathogens Research Unit, Eastern Regional Research Center, Agricultural Research Service, United States Department of Agriculture, 600 East Mermaid Lane, Wyndmoor, PA 19038, USA; gretchen.dykes@usda.gov (G.E.D.); yanhong.liu@usda.gov (Y.L.); jack.gunther@usda.gov (N.W.G.IV); katrina.counihan@usda.gov (K.L.C.); joe.lee@usda.gov (J.L.); joseph.capobianco@usda.gov (J.A.C.); 2 Foreign Arthropod-Borne Animal Disease Research Unit, National Bio and Agro-Defense Facility, Agricultural Research Service, United States Department of Agriculture, Manhattan, KS 66502, USA; siddhartha.kanrar@usda.gov

**Keywords:** *Campylobacter*, plasmid, genome sequencing, mobile genetic element, horizontal gene transfer, antibiotic resistance, virulence, foodborne pathogen

## Abstract

*Campylobacter* is one of the leading bacterial causes of gastroenteritis worldwide. It frequently contaminates poultry and other raw meat products, which are the primary sources of *Campylobacter* infections in humans. Plasmids, known as important mobile genetic elements, often carry genes for antibiotic resistance, virulence, and self-mobilization. They serve as the main vectors for transferring genetic material and spreading resistance and virulence among bacteria. In this study, we identified 34 new plasmids from 43 *C. jejuni* and *C. coli* strains isolated from retail meat using long-read and short-read genome sequencing. Pangenomic analysis of the plasmid assemblies and reference plasmids from GenBank revealed five distinct groups, namely, pTet, pVir, mega plasmids (>80 kb), mid plasmids (~30 kb), and small plasmids (<6 kb). Pangenomic analysis identified the core and accessory genes in each group, indicating a high degree of genetic similarity within groups and substantial diversity between the groups. The pTet plasmids were linked to tetracycline resistance phenotypes in host strains. The mega plasmids carry multiple genes (e.g., *aph*(3’)-III, type IV and VI secretion systems, and type II toxin–antitoxin systems) important for plasmid mobilization, virulence, antibiotic resistance, and the persistence of *Campylobacter*. Together, the identification and comprehensive genetic characterization of new plasmids from *Campylobacter* food isolates contributes to understanding the mechanisms of gene transfer, particularly the spread of genetic determinants of virulence and antibiotic resistance in this important pathogen.

## 1. Introduction

*Campylobacter* causes millions of cases of foodborne illnesses annually, imposing a significant economic burden worldwide. Of the 33 species and subspecies in the *Campylobacter* genus, *C. jejuni* and *C. coli* are the most significant in illness, responsible for nearly all human infections (http://www.who.int/news-room/fact-sheets/detail/campylobacter, accessed on 1 December 2024). A high prevalence of *Campylobacter* spp. has been reported in poultry and other meat products. Consumption of undercooked meat or cross-contaminated food is considered the primary cause of *Campylobacter* infection in humans [1,2].

*Campylobacter* spp. carry and transmit mobile genetic elements such as plasmids, phages, and transposons, facilitating the transfer of genetic information between different strains and species. Plasmids in *Campylobacter* often harbor antibiotic resistance genes, such as the pTet plasmid containing *tetO*, which encodes a ribosomal protection protein which prevents tetracyline from binding to ribosomes, thereby conferring tetracycline resistance in bacteria. Moreover, the *aph*(3’)-III gene encodes aminoglycoside-phosphotransferase, which confers resistance to aminoglycoside antibiotics such as kanamycin and streptomycin. Both *tetO* and *aph*(3’)-III have been identified in plasmids and chromosomes in *Campylobacter*. The spread of *tetO* and *aph*(3’)-III can be facilitated by plasmid transfer or transposon integration into other strains [3].

Many *Campylobacter* plasmids, including pVir, contain virulence-associated genes that enhance bacterial invasion, colonization, and survival in host cells [4]. The type IV secretion system (T4SS) is a common plasmid-born virulence factor consisting of a diverse set of genes within the *virB* and *virD* operons, encoding VirD4, VirB2, VirB4, VirB5, VirB6, VirB7, VirB8, VirB9, VirB10, VirB11, VirB12, TraG, IcmF, and Imp-like proteins. The T4SS facilitates the self-transfer of conjugative plasmids from donor to recipient cells and contributes to bacterial pathogenicity by delivering effector molecules across bacterial membranes into host cells [5].

Another important virulence factor located in plasmids or pathogenicity islands integrated into chromosomes is the type VI secretion system (T6SS). The gene cluster encoding the T6SS consists of at least 13 core components (TssA-TssM, Hcp, and VgrG) and forms a contractile T4 bacteriophage tail-like transmembrane structure. The T6SS facilitates host–pathogen interactions, delivers effector proteins to target or host cells, and induces cytotoxicity in red blood cells [6]. A recent study reported that 24.8% of *Campylobacter* genomes contain a T6SS [4].

In addition to virulence and antibiotic resistance genes, *Campylobacter* plasmids carry self-maintenance genes for replication, conjugation, mobilization, and adaptation. Through plasmid-mediated horizontal gene transfer (HGT), genetic determinants of resistance and virulence can spread to other strains or species, leading to the emergence of new pathogenic strains. Moreover, plasmid conjugation and self-mobilization between bacterial populations increase genetic diversity and adaptability in *Campylobacter*, promoting the pathogens’ survival in various environmental conditions and thus making intervention and control more challenging [7,8].

Considering that multiple plasmids can co-exist in the same strain but differ in their ability to conjugate or be mobilized between strains/species, it is important to investigate how plasmids differ in their genetic content. In addition, since poultry and meat products are the main reservoirs for *Campylobacter*, it is important to determine if *Campylobacter* strains from different food companies or isolation sources carry the same virulence and antibiotic resistance genes. A previous work characterized *Campylobacter* plasmids from retail meats; however, in that study, strainss were primarily isolated in Tulsa, Oklahoma, USA [9]. It is currently unknown if similar plasmid profiles exist in other areas of the United States, such as in the Mid-Atlantic region. Understanding how these mobile genetic elements circulate in *Campylobacter* populations in food clarifies the impact of plasmids on the dissemination and persistence of antibiotic resistance and virulence [8].

In this study, we applied long-read and short-read genome sequencing techniques to identify new plasmids in *Campylobacter* food isolates, compare the genetic relatedness and diversity of the plasmids, and predict their functions. The potential transfer of virulence and antibiotic resistance genes between strains and species was inferred from the phylogeny and pangenomic analysis of the plasmids. The results of this study enhance our understanding of how the pathogens develop and spread resistance and virulence traits and adapt to different environments, thereby assisting in the development of effective strategies to control their spread.

## 2. Materials and Methods

### 2.1. Sample Preparation

Each *Campylobacter* strain was isolated from an independent package of chicken meat, chicken liver, or beef liver acquired from local retailers or poultry processing plants in the Mid-Atlantic area in the USA from 2011 to 2023, using previously established methods [10]. Briefly, 450 g meat or liver was massaged with 250 mL buffered peptone water (BPW, Bio-Rad Laboratories Inc., Hercules, CA, USA) in a filtered stomacher bag. The liquid from the filtered side of the stomacher bag was collected and centrifuged (10,000× *g* for 10 min). Following centrifugation, the supernatant was discarded, and the pellet was resuspended and enriched in Bolton broth (Remel Inc., Lenexa, KS, USA) with horse blood and selective supplements (cefoperazone, trimethoprim, vancomycin, and cycloheximide) under microaerobic conditions (5% O_2_, 10% CO_2_, and 85% N_2_) at 42 °C for 24 hrs. Due to the high motility of *Campylobacter* spp., passive filtration of the enrichment onto Brucella agar (Becton, Dickinson and Company, Franklin Lakes, NJ, USA) was used for strain isolation. The enrichment culture (20 µL) was spotted on a 0.45 µm cellulose acetate filter on top of a Brucella agar plate. The filter and drop of enrichment culture were allowed to rest for 15 min (to provide sufficient time for mobile *Campylobacter* spp. to traverse the filter); then, the filter was removed, and the Brucella plate was incubated under microaerobic conditions at 42 °C for 24 h. After re-streaking for purified colonies, the genus and species of the isolates were determined by the multiplex quantitative polymerase chain reaction (qPCR) assay previously developed for the identification and differentiation of *C. jejuni* and *C. coli* [11]. Purified colonies were stored in DMSO stock at −80 °C following purification and re-cultured on Brucella agar plates and, finally, liquid Brucella media prior to sequencing, resulting in no more than two transfers of purified colonies prior to sequencing or phenotypic assays.

### 2.2. Genome Sequencing, Assembly, and Plasmid Identification

Genomic DNA was extracted using the Qiagen genomic tip 100/G kit (Valencia, CA, USA) and quantified with a Qubit 3.0 fluorometer (Thermo Fisher Scientific, Waltham, MA, USA), following the manufacturers’ instructions. Whole genomes were sequenced using Illumina MiSeq (San Diego, CA, USA), Pacific Biosciences (PacBio, Menlo Park, CA, USA) RSII, and/or PacBio Sequel. In addition, several *Campylobacter* genomes previously assembled [12] using PacBio long reads with Canu v2.2 [13] were incorporated. Initial assembly was performed using PacBio data with the arguments ‘corMhapSensitivity = high’, ‘corMinCoverage = 100’, and ‘genomeSize = 1.83 m’. In some cases where a chromosome size contig was not assembled, a less stringent minimum coverage parameter, ‘corMinCoverage = 0’, was used. Following assembly, contig overhangs were trimmed, and the contigs were reoriented using Circlator v1.5.5 [14].

In some instances where reorientation and trimming failed, sequencing errors in the assembled contigs were corrected using Illumina MiSeq reads. First, MiSeq reads were mapped to Canu assembled contigs using BWA v0.7.17-r1188 [15]. Then, errors were corrected using Pilon v1.22 [16] with the parameters “--fix all” and “--mindepth 0.5”. Pilon correction was repeated until no errors were reported. Finally, the contigs were trimmed and reoriented using Circlator v1.5.5.

For each strain, the contig closest in size to previously sequenced *Campylobacter* chromosomes (~1.6–1.8 Mbp) was annotated using RAST [17,18] to ensure that the origin was set to *dnaA*; three copies of rRNA subunits (23S, 16S, and 5S rRNA) and minimal repeats were present in the chromosome. Smaller contigs were examined for the potential presence of plasmids.

**Figure 1:**
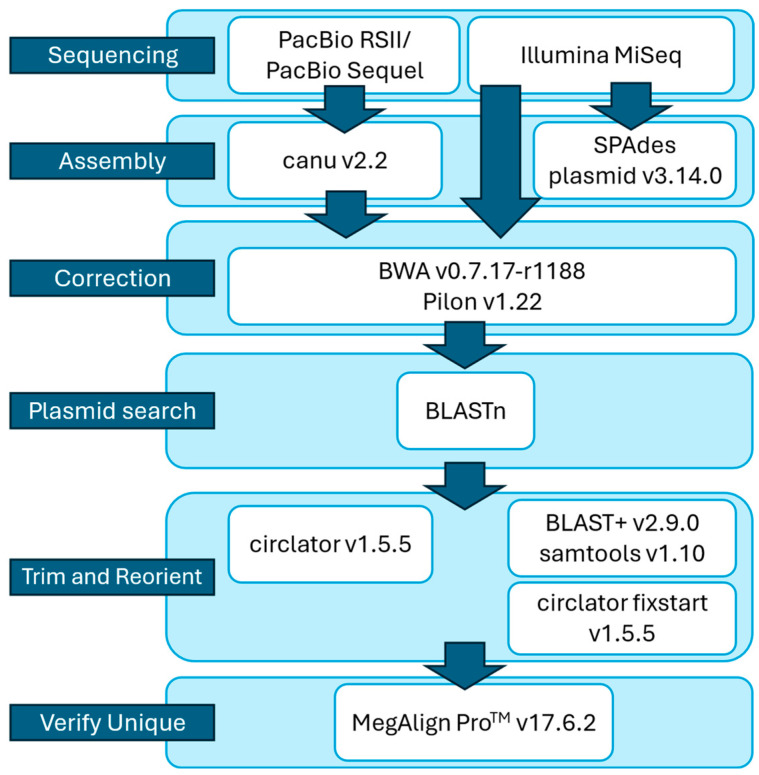
Schematic representation of the plasmid assembly workflow.

To search for plasmids in whole-genome sequences, all small contigs (<1 Mbp) assembled from Illumina MiSeq reads with SPAdes v3.14.0 [19] using the ‘--plasmid’ argument and PacBio reads assembled with Canu v2.2 were queried with BLAST [20] for nucleotide similarity to known plasmids in GenBank (www.ncbi.nlm.nih.gov/genbank/). Small contigs were considered plasmid candidates if most of the top BLAST hits aligned to plasmid DNA. Overhang on potential plasmids was trimmed using BLAST+ v2.9.0 [21] and samtools v1.10 [22] or Circlator v1.5.5 [14]. Next, potential plasmids were reoriented with Circlator v1.5.5 using the fix-start function [14]. Finally, for each strain, the potential plasmids were aligned to the chromosome and each other using MegAlign Pro^TM^ (“MegAlign Pro”, n.d., Madison, WI, USA) [23] to ensure that the final plasmids of a strain were not redundant sequences (Figure 1). This pipeline verified the integrity of the putative plasmids by ensuring that the top BLAST hits for each plasmid were other plasmid sequences (rather than chromosomal DNA) and by aligning plasmids to each other within a strain to avoid redundant sequences. Furthermore, we ran Circulator to trim overhang, which indicates that the plasmid was intact and not a fragment of a larger plasmid. Small plasmids were further verified by agarose gel electrophoresis.

### 2.3. Plasmid Pangenome and Phylogeny

To determine which genes were shared among multiple plasmids, we constructed a *Campylobacter* plasmid ‘pangenome’ with OrthoMCL v0.0.8 [24] using RAST-tk annotations [25] on the KBase Server [26]. In addition to the newly assembled plasmids reported here, we also incorporated previously published plasmids from our laboratory and reference plasmids from GenBank (Table 1). Heatmaps depicting gene presence/absence and the number of shared genes among plasmids were generated in R v4.4.0 [27] using ggplot2 and viridis [28,29]. To investigate the relatedness of different plasmid clusters, we constructed reference sequence-based alignment phylogenetic trees using the REALPHY web server [30]. Reference sequences are listed in Table 1. Where there were multiple references in a cluster, we merged the final alignments. The small plasmids had higher sequence variation than the other plasmid clusters; therefore, the small plasmid phylogenetic trees were built with RAXML-NG [31] using multiple-sequence alignment from Muscle [32] in MegAlign (“MegAlign Pro”, n.d., Madison, WI, USA). Trees were visualized and annotated using Iroki [33].

To understand the replicon types and mobility of the plasmids, we characterized all plasmids with the mob-typer tool in the mob-suite v3.1.9 [34,35].

**Table 1:** List of the plasmids identified in *C. jejuni* and *C. coli* isolates.

Strain and Species	Source	Plasmid Name	Size (bp)	%GC	Accession No.	Reference
*C. jejuni* YH001	Veal livers	pCJP001-1	46,524	29.74	CP173351	This work
*C. jejuni* YH001	Veal livers	pCJP001-2	4354	30.57	CP173352	This work
*C. jejuni* YH002	Calf livers	pCJP002	45,904	29.20	CP020775	[36]
*C. jejuni* YH016	Calf livers	pCJP016	29,736	28.21	CP157938	This work
*C. jejuni* YH018	Calf livers	pCJP018-1	46,524	29.74	CP172373	This work
*C. jejuni* YH018	Calf livers	pCJP018-2	4366	30.85	CP172374	This work
*C. jejuni* YH019	Beef livers	pCJP019-1	46,275	28.99	CP172369	This work
*C. jejuni* YH019	Beef livers	pCJP019-2	30,011	28.18	CP172370	This work
*C. jejuni* YH019	Beef livers	pCJP019-3	4367	30.82	CP172371	This work
*C. jejuni* YH020	Veal livers	pCJP020	37,426	27.78	CP172367	This work
*C. jejuni* YH024	Calf livers	pCJP024-1	45,034	29.55	CP172359	This work
*C. jejuni* YH024	Calf livers	pCJP024-2	4366	30.85	CP172360	This work
*C. jejuni* YH025	Calf livers	pCJP025	41,594	28.51	CP172357	This work
*C. jejuni* YH026	Calf livers	pCJP026	44,973	29.12	CP172355	This work
*C. jejuni* YH027	Calf livers	pCJP027	46,515	29.72	CP172353	This work
*C. jejuni* YH029	Beef livers	pCJP029	16,920	28.27	CP172350	This work
*C. jejuni* (S33Cj) YH010	Chicken thighs	pCJS010 (pCjS33)	40,686	28.49	CP131443	[37]
*C. jejuni* (S36Cj) YH011	Chicken thighs	pCJS011 (pCjS36)	86,827	26.03	CP131441	[37]
*C. jejuni* YH014	Chicken livers	pCJS014-1	47,468	30.28	CP172377	This work
*C. jejuni* YH014	Chicken livers	pCJS014-2	43,660	29.00	CP172378	This work
*C. jejuni* YH021	Chicken breasts	pCJS021	43,177	28.96	CP172365	This work
*C. jejuni* YH022	Chicken thighs	pCJS022	48,862	28.64	CP172363	This work
*C. coli* YH502	Chicken drumsticks	pCOS502	125,964	28.11	CP018901	[38]
*C. coli* YH503	Chicken drumsticks	pCOS503-1	108,453	26.15	CP025282	[12]
*C. coli* YH503	Chicken drumsticks	pCOS503-2	5401	32.85	CP173353	This work
*C. coli* YH504	Chicken drumsticks	pCOS504	110,357	26.02	CP091645	[12]
*C. coli* YH504	Chicken drumsticks	pCOS504-2	5401	32.85	CP173354	This work
*C. coli* YH506	Chicken wings	pCOS506	5402	30.53	CP172398	This work
*C. coli* YH507	Chicken livers	pCOS507-1	150,434	27.53	CP172393	This work
*C. coli* YH507	Chicken livers	pCOS507-2	37,224	25.96	CP172394	This work
*C. coli* YH507	Chicken livers	pCOS507-3	29,068	29.33	CP172395	This work
*C. coli* YH510	Chicken livers	pCOS510-1	117,204	28.20	CP172388	This work
*C. coli* YH510	Chicken livers	pCOS510-2	38,174	25.80	CP172389	This work
*C. coli* YH511	Chicken livers	pCOS511	30,429	27.88	CP172386	This work
*C. jejuni* RM1246-ERRC	Human	pRM1246_ERRC	45,197	29.14	CP022471	[39]
*C. jejuni* RM3194	Human	pRM3194	81,079	25.99	CP014345	[40]
*C. jejuni* 81-176	Human	pTet *	45,025	29.09	CP000549	N/A
*C. jejuni* 81-176	Human	pVir *	37,473	25.89	CP000550	N/A
*C. coli* CVM N17C336	Chicken breasts	pN17C336-1 *	146,302	27.99	CP169431	N/A
*C. coli* CVM N17C264	Chicken breasts	pN17C264-2 *	39,356	26.18	CP169460	N/A
*C. jejuni* NADC 20827	Turkey	p20827S *	4366	30.83	CP045047	[41]
*C. coli* CC20JX12	Meat	pCC20JX12-5K *	5363	31.51	CP109816	N/A
*C. coli* 2014D-0261	Not reported	p2014D0261-1 *	52,384	28.41	CP059367	N/A
*C. jejuni* AR-0413	Not reported	pAR-0413-2 *	25,131	28.47	CP044172	N/A
*C. jejuni* PNUSC002710	Not reported	pNUSAC002710-2 *	28,157	28.03	CP132117	N/A
*C. coli* XK3140	Chicken liver	pCCDM140S *	26,812	29.28	MH634990	[9]
*C. jejuni* RM1477	Human	pRM1477 *	28,220	27.93	CP071588	[42]

* Reference plasmids used for analyses. N/A indicates not applicable. These plasmids are available in GenBank, but there is no publication noted on the database entry to cite.

### 2.4. Tetracycline Resistance Testing

Strain resistance to tetracycline was assessed using the Clinical Laboratory Standards Institute (CLSI, 2015) broth microdilution technique with Sensititre plates (ThermoFisher Scientific, Clevland, OH, USA), as described in Ghatak et al., in 2020 [38].

## 3. Results and Discussion

### 3.1. In Silico Identification of Large and Small Plasmids in Campylobacter Food Isolates

In this study, we identified 34 new plasmids from 32 *C. jejuni* and 11 *C. coli* genomes using PacBio HiFi long-read sequencing and Illumina Miseq short-read sequencing. The de novo-assembled plasmid contigs were confirmed through a BLAST search for nucleotide similarity to known plasmids in GenBank. Plasmids were verified to ensure that there was no similarity to their chromosome or other plasmid sequences in the same strain. All plasmid-carrying strains were independent *Campylobacter* isolates from individual packages of fresh chicken meat, chicken liver, or beef liver collected from different vendors or processors between 2011 and 2023. Table 1 summarizes the host strain, source, sequence, and assembly information of the plasmids identified in *Campylobacter* food isolates.

Out of 43 *Campylobacter* isolates, 25 were found to carry 1–3 plasmids per strain. The plasmids ranged in size from 4.3 to 150.4 kb. All the mega plasmids were assembled from the long-read sequences, whereas small plasmids were identified only from short-read sequence assemblies, due to the 10 kb size cutoff during PacBio library preparation. The average %GC content of the plasmids was 28.8%, which is lower than that of host chromosomes (~30–31%). The size and GC content of the new plasmids correspond well to known *Campylobacter* plasmids in the NCBI database.

### 3.2. Pangenomic Analyses of the Conserved Core Genes and Diversity of the Plasmids

**Figure 2:**
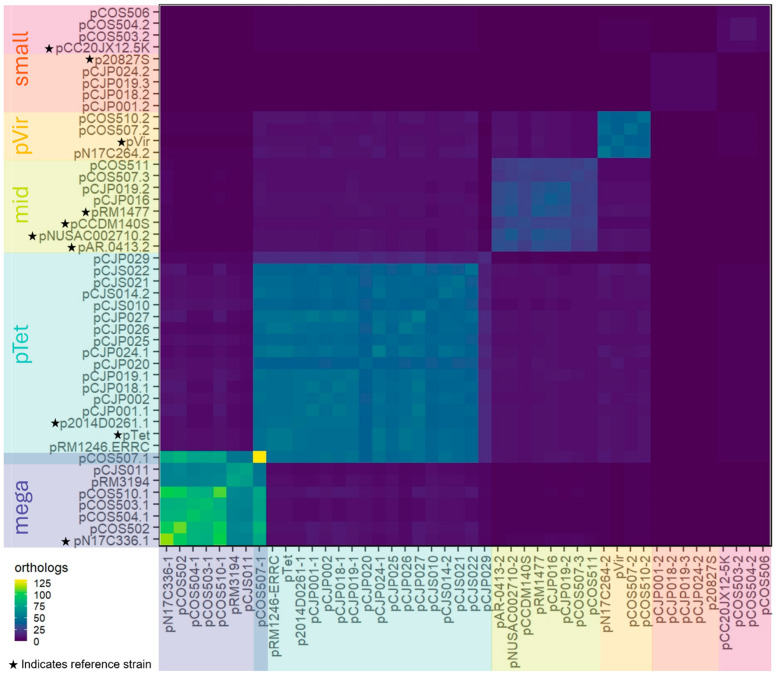
Heatmap showing the number of shared genes across the plasmid pangenome: the higher the number of shared genes, the more yellow the cell; the less shared genes, the more purple the cell. Plasmid labels are shaded according to our manual clustering groups. Reference strains are indicated with a star on the y-axis.

To determine the relatedness of the plasmids, we conducted a pangenomic analysis using OrthoMCL v0.0.8 [24]. By comparing the number of genes shared among the plasmids, we categorized the plasmids into five groups (Figure 2): mega plasmids (>80 kb), pTet (containing the *tetO* gene), mid-sized plasmids (~30 kb), pVir, and small plasmids (<6 kb). The heatmap in Figure 2 shows that the mega plasmids have the highest number of shared genes, whereas the small plasmids have the least shared genes. No genes were shared among all 46 plasmids (including references). However, 275 genes were shared between 2 and 33 plasmids (non-core genes), and 318 were present only in a single plasmid (Supplementary Table S1).

Functional annotation with RASTtk (Rapid Annotations using Subsystems Technology toolkit) predicted that plasmid genes are involved in antimicrobial resistance, virulence, and horizontal gene transfer between strains or species (Supplementary Table S2).

#### 3.2.1. pTet

**Figure 3:**
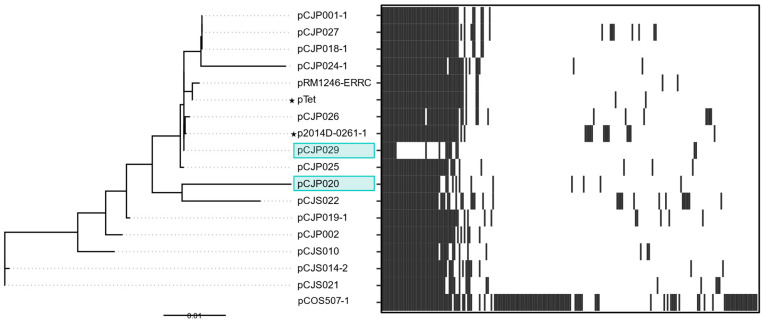
Phylogenetic tree and presence/absence chart of genes contained in pTet plasmids. In the phylogenetic tree, reference pTet plasmids are indicated with a star; plasmids that are clustered with pTet but only contain chromosomal *tetO* are highlighted in teal. Solid lines in the phylogenetic tree indicate distance between plasmids. Dotted lines are for esthetic purposes to ease in the interpretation of the figure, given the aligned tip labels. In the presence/absence chart, the presence of gene clusters in the plasmid is indicated in dark gray, while the absence of genes is indicated in white; the location on the x-axis does not indicate the location on the plasmid. The scale bar for the phylogenetic tree indicates the distance in units of nucleotide substitutions per site.

Bacterial plasmids often contain genes encoding antibiotic resistance, which can rapidly spread between different strains and species. We found that 14 out of 36 plasmids (36%, excluding references, including previously published plasmids) contained the tetracycline resistance gene *tetO*, indicating that pTet was the most prevalent type of plasmid in our *Campylobacter* food isolates, which is consistent with other reports [9,43]. In the pangenomic analysis, 16 plasmids from both *C. jejuni* and *C. coli* isolates clustered together and close to the well-studied pTet from *C. jejuni* 81-176 (Figure 3). This indicates that the pTet plasmids reported here are genetically related to each other, suggesting that pTet might be obtained through horizontal gene transfer events between different strains and species. This is supported by the core pTet genome, which contains T4SS genes and conjugative transfer genes, and the non-core genome which contains tetracycline and kanamycin resistance genes. Two plasmids, pCJP020 and pCJP029, shared a number of genes with the rest of the pTet plasmid group but did not contain *tetO.* Although pCJP029 shared more genes with pTet group plasmids than any other groups, far fewer genes were shared between pCJP029 and pTet group plasmids than between plasmids within the pTet group.

To investigate the phylogenetic relatedness of the pTet cluster, we constructed a tree of pTet sequences aligned to the reference plasmids pTet and p2014D0261-1 (Figure 3). The plasmid pCOS507-1 (150.4 kb) was excluded from the tree because it was significantly larger than other pTet plasmids (<48.8 kb). Although pCJP020 and pCJP029 did not contain *tetO*, both were closely related to other pTet plasmids and did not cluster as an outgroup. A BLAST search confirmed that, while *tetO* was absent from the pCJP020 and pCJP029 plasmids, *tetO* was located in the chromosome. This suggests that *tetO* may have been lost from the plasmid and subsequently integrated into the chromosome. Two additional strains, YH002 and YH019, also contained *tetO* in both their chromosomes and pTet plasmids. When analyzed phylogenetically, the chromosomal *tetO* amino acid sequences clustered together and independently from plasmid *tetO* sequences within the same strain (Supplementary Figure S1). This suggests that some *tetO* genes or plasmids could be more suited for chromosomal integration. Given the presence of *tetO* in the chromosome and plasmid in multiple strains, as well as the high proportion of pTet plasmids among the sequenced genomes, these results indicate a strong selective advantage for *tetO* maintenance and suggest frequent horizontal gene transfer events.

Across the pTet plasmids, several core genes (present in all 16 pTet plasmids) were involved in horizontal gene transfer and antibiotic resistance (Supplementary Table S3). Eleven out of thirty-three core gene clusters belonged to Type IV secretion systems (T4SS), including clusters encoding VirB9, VirB5, VirB10, VirB2, VirB6, VirB7, VirB8, VirD4, VirB11, VirB3, and VirB4. In addition, several annotated core genes were involved in conjugative transfer, including *traG*, *traR*, and *traQ*. One core gene was a site-specific recombinase in the resolvase family. Antibiotic resistance genes were identified as non-core genes, including *tetO*, which was present in 14 of the pTet plasmids, and *aph*(3’)-III (conferring resistance to aminoglycosides), which was present in 6 of the pTet plasmids and 3 additional mega plasmids.

To determine the resistance phenotype of the pTet-containing strains, we assessed tetracycline resistance of all the isolated strains (Table 2). All pTet-carrying strains, including both *C. jejuni* and *C. coli* species, were resistant to tetracycline, with minimum inhibitory concentrations (MICs) greater than or equal to 4 µg/mL, and with most strains greater than or equal to 64 µg/mL. This includes the strains harboring plasmids pCJP020 (YH020) and pCJP029 (YH029), which clustered with the pTet plasmids but did not contain plasmid *tetO* and instead carried chromosomal *tetO*.

The consistency between genotype and phenotype demonstrates that the *tetO* gene, whether located in pTet plasmids or the chromosomes, contributes to the tetracycline resistance of *Campylobacter* strains. The observed high rate of tetracycline resistance in *Campylobacter* isolates from meat products could be related to the use of tetracycline as a growth promoter in animal feed [44], raising concerns about the transmission of antimicrobial resistance through food sources. Low-level antimicrobial resistance may arise from exposure to low-dose antibiotics [45], and low-dose exposure of tetracycline may explain the prevalence of pTet plasmids across *Campylobacter* isolates, especially in the strains harboring pTet plasmids with tetracycline MICs of 4 µg/mL. In addition, high plasmid stability may contribute to the observed high frequency of strains with pTet plasmids.

#### 3.2.2. pVir

**Figure 4:**
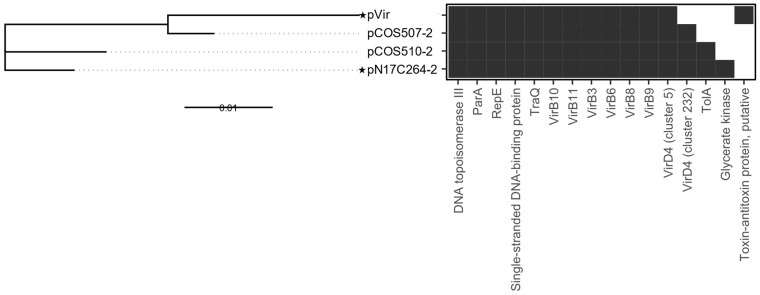
Phylogenetic tree and presence/absence chart of genes in pVir plasmids. In the phylogenetic tree, reference pTet plasmids are indicated with a star. Solid lines in the phylogenetic tree indicate distance between plasmids. Dotted lines are for esthetic purposes to aid the interpretation of the figure given the aligned tip labels. In the presence/absence chart, the presence of gene clusters in the plasmid is indicated in dark gray, while the absence of gene clusters is indicated in white; location on the x-axis does not indicate location on the plasmid. The scale bar for the phylogenetic tree indicates the distance in units of nucleotide substitutions per site.

pVir was initially identified in the clinical *C. jejuni* strain 81-176 and is believed to contribute to bloody diarrhea in *C. jejuni* enteritis [46]. pVir infrequently occurs in *Campylobacter* [43]. In this study, only two *C. coli* plasmids (pCOS507-2 and pCOS510-2) from chicken liver isolates were clustered in the same group as pVir based on the number of shared genes (Figure 2). These plasmids clustered closely to pVir in the phylogenetic analysis (Figure 4). pCOS507-2 and pCOS510-2 share 99% sequence homology to pVir and share 35 core genes and 19 non-core genes (Supplementary Table S1). pVir group plasmids possess the same core set of virulence factors, including T4SS: Vir B3, VirB4, VirB6, VirB8, VirB9, VirB10, VirB11 (core), and VirD4 (non-core, Supplementary Table S4). In addition to the T4SS, all pVir plasmids contained the plasmid conjugative transfer protein TraQ and the plasmid partitioning protein ParA. Together, these results suggest a high virulence potential of these plasmid-harboring strains from food.

#### 3.2.3. Small Plasmids (<6 kb)

**Figure 5:**
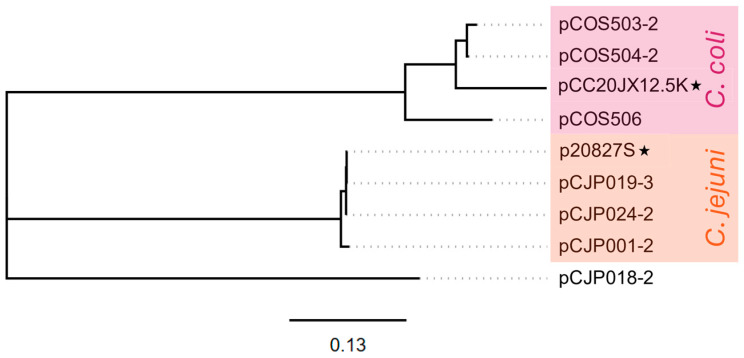
Phylogenetic tree of multiple-sequence alignment of small plasmid sequences. Reference sequences are indicated with a star. Solid lines in the phylogenetic tree indicate distance between plasmids. Dotted lines are for esthetic purposes to aid the interpretation of the figure given the aligned tip labels. Small plasmids are clustered into three groups, one composed of *C. coli* sequences (highlighted in pink), one composed of *C. jejuni* sequences (highlighted in orange), and a singular *C. jejuni* sequence. The scale bar for the phylogenetic tree indicates the distance in units of nucleotide substitutions per site.

The small plasmids (<6 kb) shared few genes with other plasmids, and all but one small plasmid co-existed with large plasmids in the same host strains. Pangenomic analysis clustered the small plasmids into two groups, each with only five shared core genes (Figure 2, Supplementary Table S5). Phylogenetically (based on multiple-sequence alignment), the small plasmids formed three separate groups, consistent with the pangenome groups (Figure 5). Group 1 contained only plasmids from *C. coli*, including pCOS503-2, pCOS405-2, pCOS506, and the reference pCC20JX12-5K; group 2 contained only plasmids from *C. jejuni*, including pCJP019-3, pCJP024-2, pCJP001-2, and the reference p20827S. Finally, one plasmid, pCJP018-2, clustered separately from the *C. coli* and *C. jejuni* small-plasmid groups. This may indicate that small plasmids are more likely to be species-specific than larger plasmids, perhaps because they are more reliant on host/larger plasmid replication machinery than larger plasmids, which may possess more self-replication genes.

To better understand whether small plasmids are related to the host species in *C. coli* and *C. jejuni*, we constructed a multiple-sequence alignment tree of all *C. coli* and *C. jejuni* small plasmids (<6 kb) available on NCBI and predicted their mobility using MOB-suite v3.1.9 [34,35] (Supplementary Figure S2). Plasmids from different species were found in the same clades, though some clades were composed of mostly or entirely *C. coli* or *C. jejuni* plasmids. Therefore, it is unlikely for small plasmids to have high specificity for a single species. However, the majority of small plasmids were found to be non-mobilizable (79%), indicating that most small plasmids rely on the host or larger plasmid machinery to spread to other hosts.

To better understand how small plasmids may rely on host/large plasmid machinery, we predicted the mobility and type of plasmids using the MOB-suite software v3.1.9 [34,35]. None of the small plasmids were predicted to be conjugative, further supporting the idea that small plasmids rely on larger plasmid/host machinery to spread (Table 3). The predicted mobility of the plasmids correlated with their phylogenetic grouping, with *C. jejuni* small plasmids being categorized as mobilizable whereas *C. coli* small plasmids were categorized as non-mobilizable. Mobilizable plasmids contain a relaxase and the origin of transfer (*oriT*) but lack a mate-pair formation marker and can be transferred with the help of a conjugative plasmid, while non-mobilizable plasmids lack a relaxase and *oriT* and cannot be moved via conjugation [34]. All *C. jejuni* (but not *C. coli*) small plasmids co-existed with a conjugative pTet plasmid (Table 3), which may facilitate the horizontal transfer of small mobilizable plasmids. On the other hand, *C. coli* small plasmids were found to exist alongside non-mobilizable mega plasmids or alone, indicating the inability to transfer horizontally.

The analysis of replicon types with MOB-suite demonstrated that the small plasmids had distinct replicon types from the large plasmids, with the *C. jejuni* small plasmids categorized as cluster 795 replicons and the *C. coli* small plasmids categorized as cluster 896 replicons (Table 3). The mega and mid plasmids were not assigned a replicon cluster, but the pTet plasmids were typically cluster 475, and the pVir plasmids were cluster 1502. Therefore, small and large plasmids do not share the same replication systems, supporting the idea that distantly related plasmids tend to be compatible with each other in the same bacterial cell [47].

Except for limited plasmid self-maintenance genes, no other functions were predicted in the sequences of small plasmids, which may reflect limitations in current annotation methods and/or databases for identifying small protein-encoding genes and functional RNA genes. The only annotated core genes were found in group 1 (*C. coli*) small plasmids, all of which shared a site-specific recombinase and the putative replication protein RepE.

#### 3.2.4. Mega Plasmids (>80 kb)

**Figure 6:**
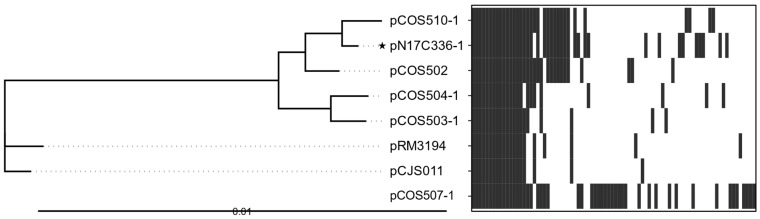
Phylogenetic tree of multiple-sequence alignment and presence/absence chart of genes in mega plasmid sequences. Reference sequences are indicated with a star. Solid lines in the phylogenetic tree indicate distance between plasmids. Dotted lines are for esthetic purposes to aid the interpretation of the figure given the aligned tip labels. In the presence/absence chart, the presence of gene clusters in the plasmid is indicated in dark gray, while the absence of gene clusters is indicated in white; the location on the x-axis does not indicate the location on the plasmid. The scale bar for the phylogenetic tree indicates the distance in units of nucleotide substitutions per site.

The group of mega plasmids (>80 kb) found in *C. jejuni* and *C. coli* isolates shared 42 conserved core genes and had high genetic similarity (Supplementary Table S1, Figure 6). Most of the annotated mega plasmid core genes were involved in the type VI secretion system (T6SS), including ImpA, ImpB, ImpC, ImpG, ImpH, ImpJ, ImpK, IcmF, Hcp, and vasD (Supplementary Table S6). Non-core genes were involved in self-maintenance, antibiotic resistance, and conjugation. Three (42%) of the mega plasmids contained aminoglycoside O-phosphotransferase encoded by *aph*(3’)-IIIa, which confers resistance to aminoglycoside antibiotics. However, of the seven mega plasmids, only pCOS507-1 contained *tetO*, which confers tetracycline resistance to the host strain *C. coli* YH507. Five (71%) of the mega plasmids contained the type II toxin–antitoxin system death-on-curing protein Doc. Type II toxin–antitoxin systems were reported to be involved in bacterial pathogenesis by maintaining virulence plasmids and inducing the expression of virulence-associated genes [48].

We found that the mega plasmids were categorized as mobilizable or non-mobilizable plasmids, contrasting with the mid, pVir, and pTet plasmids, which were all categorized as conjugative (Table 3). However, we noted multiple genes involved in conjugative transfer, including IncF plasmid conjugative transfer protein TraG (core, 100%), TrsK-like protein (29%), and VirB6 (42%), were present in the mega plasmids. Other conjugative transfer proteins were observed more sporadically in the mega plasmids (Supplementary Table S6). In addition, mega plasmids that were mobilizable encoded a MOBQ-type relaxase, as opposed to the MOBP-type relaxase which was found in all pVir, pTet, mid, and small plasmids.

The presence of T6SS, antibiotic resistance, and toxin–antitoxin genes in the mega plasmids suggests significant potential for antibiotic resistance and virulence in the host strains, as well as the possible spread of pathogenicity to other strains via plasmid conjugation or mobilization. These findings align with recent studies of pCJDM202/pCJDM67L, a *Campylobacter* mega plasmid containing tetracycline resistance genes, conjugative transfer (T4SS), and the Type VI secretion system (T6SS). pCJDM202/pCJDM67L increased cytotoxicity to red blood cells when transferred to its recipient strain through conjugation [9].

#### 3.2.5. Mid Plasmids (~30 kb)

The group of mid plasmids (~30 kb) in *Campylobacter* displayed high similarity and shared 20 conserved core genes (Supplementary Table S1). Most of the annotated mid plasmid core genes were the components of RP4-specific conjugative transfer apparatus (TrbC, TrbD, TrbE, TrbF, TrbG, TrbI, TrbJ, TrbL, and TrbM) and T4SS (VirB11, VirB1, VirD4, VirB3, and VirB5), which are involved in plasmid conjugative transfer (Supplementary Table S7). In addition, the mid plasmids were categorized as conjugative (Table 3), consistent with the annotated functions of mid plasmid genes.

## 4. Conclusions

This study identified 34 new plasmids from *Campylobacter* food isolates, uncovering their complete sequences and functional annotations. Comprehensive genomic analysis revealed critical genes and gene operons associated with antibiotic resistance, virulence, and the transfer of genetic elements within *Campylobacter*. Notably, the presence of *tetO* and aminoglycoside resistance genes underscores the role of these plasmids in mediating multidrug resistance, a significant challenge in both clinical and agricultural settings. The identification of Type IV and Type VI secretion systems (T4SS and T6SS) further highlights the contribution of these plasmids to pathogenicity and their potential role in facilitating the horizontal transfer of virulence factors between strains.

Phylogenetic and pangenomic studies provided insight into the genetic relatedness of plasmids within groups, while demonstrating diversity between groups. These findings are significant, advancing our understanding of the genetic basis of bacterial evolution through the transfer of genetic elements and the spread of antibiotic resistance and virulence factors among pathogens. The described bioinformatics workflow for the identification and genetic characterization of large and small plasmids in *Campylobacter* strains represents a valuable resource. It provides a robust framework for studying mobile genetic elements, virulence factors, and antibiotic resistance determinants in *Campylobacter* and related microorganisms. This research not only deepens scientific knowledge but also supports the development of targeted interventions to eliminate the spread of antimicrobial resistance and enhance food safety.

## Figures and Tables

**Table 2:** Tetracycline MIC for *Campylobacter* strains containing *tetO*.

Strain	Plasmids	Tetracycline MIC (µg/mL)	*tetO* Location
*C. jejuni* YH014	pCJS014-2	>64	pTet
*C. jejuni* YH019	pCJP019-1, pCJP019-2, pCJP019-3	>64	pTet and chromosome
*C. jejuni* YH020	pCJP020	>64	chromosome
*C. jejuni* YH025	pCJP025	>64	pTet
*C. coli* YH507	pCOS507-1, pCOS507-2, pCOS507-3	>64	pTet
*C. jejuni* YH001	pCJP001-1, pCJP001-2	64	pTet
*C. jejuni* YH002	pCJP002	64	pTet and chromosome
*C. jejuni* YH018	pCJP018-1	64	pTet
*C. jejuni* YH022	pCJS022	64	pTet
*C. jejuni* YH024	pCJP024-1, pCJP024-2	64	pTet
*C. jejuni* YH027	pCJP027	64	pTet
*C. jejuni* YH029	pCJP029	64	chromosome
*C. jejuni* YH026	pCJP026	32	pTet
*C. jejuni* YH010	pCJS010	4	pTet
*C. jejuni* YH021	pCJS021	4	pTet
*C. coli* YH503	pCOS503-1, pCOS503-2	0.5	none
*C. coli* YH504	pCOS504-1, pCOS504-1	0.25	none
*C. coli* YH510	pCOS510-1, pCOS510-2	0.25	none
*C. coli* YH511	pCOS511	0.25	none
*C. jejuni* YH011	pCJS011	0.12	none
*C. coli* YH506	pCOS506	0.12	none
*C. coli* YH502	pCOS502	0.06	none

**Table 3:** Replicon, relaxase, and mobility type of *Campylobacter* plasmids.

Plasmid Name	Replicon Type	Relaxase Type	Predicted Mobility	Cluster
pCOS502	-	MOBQ	mobilizable	mega
pCOS510-1	-	MOBQ	mobilizable	mega
pN17C336-1 *	-	MOBQ	mobilizable	mega
pCJS011 (pCjS36)	-	-	non-mobilizable	mega
pCOS503-1	-	-	non-mobilizable	mega
pCOS504	-	-	non-mobilizable	mega
pRM3194	-	-	non-mobilizable	mega
pCOS507-1	rep_cluster_475	MOBP	conjugative	mega/pTet
pAR-0413-2	-	-	-	mid
pCCDM140S	-	-	-	mid
pNUSAC002710-2	-	-	-	mid
pRM1477	-	-	-	mid
pCJP016	-	MOBP	conjugative	mid
pCJP019-2	-	MOBP	conjugative	mid
pCOS507-3	-	MOBP	conjugative	mid
pCOS511	-	MOBP	conjugative	mid
p2014D0261-1	-	-	-	pTet
pCJP001-1	rep_cluster_475	MOBP	conjugative	pTet
pCJP002	rep_cluster_475	MOBP	conjugative	pTet
pCJP018	rep_cluster_475	MOBP	conjugative	pTet
pCJP019-1	rep_cluster_475	MOBP	conjugative	pTet
pCJP020	-	MOBP	conjugative	pTet
pCJP024	rep_cluster_475	MOBP	conjugative	pTet
pCJP025	-	MOBP	conjugative	pTet
pCJP026	rep_cluster_475	MOBP	conjugative	pTet
pCJP027	rep_cluster_475	MOBP	conjugative	pTet
pCJS010 (pCjS33)	-	MOBP	conjugative	pTet
pCJS014-2	-	MOBP	conjugative	pTet
pCJS021	-	MOBP	conjugative	pTet
pCJS022	-	MOBP	conjugative	pTet
pRM1246_ERRC	rep_cluster_475	MOBP	conjugative	pTet
pTet *	rep_cluster_475	MOBP	conjugative	pTet
pCJP029	rep_cluster_475	MOBP	mobilizable	pTet
pCOS507-2	rep_cluster_1502	MOBP	conjugative	pVir
pCOS510-2	rep_cluster_1502	MOBP	conjugative	pVir
pN17C264-2 *	rep_cluster_1502	MOBP	conjugative	pVir
pVir *	rep_cluster_1502	MOBP	conjugative	pVir
p20827S *	rep_cluster_795	MOBP	mobilizable	small
pCJP001-2	rep_cluster_795	MOBP	mobilizable	small
pCJP018-2	rep_cluster_795	MOBP	mobilizable	small
pCJP019-3	rep_cluster_795	MOBP	mobilizable	small
pCJP024-2	rep_cluster_795	MOBP	mobilizable	small
pCC20JX12-5K *	rep_cluster_896	-	non-mobilizable	small
pCOS503-2	rep_cluster_896	-	non-mobilizable	small
pCOS504-2	rep_cluster_896	-	non-mobilizable	small
pCOS506	-	-	non-mobilizable	small

“-” Indicates no prediction reported from MOB-suite. * Reference plasmids used for analyses.

## Data Availability

All the assembled plasmid sequences from *Campylobacter* isolates were deposited and are available in GenBank, NCBI, under the accession numbers listed in Table 1.

## Supplementary Materials

The following supporting information can be downloaded at: https://www.mdpi.com/article/10.3390/microorganisms13010206/s1.
