# Supplementary material for: Comparative Genomic Analysis of Campylobacter Plasmids Identified in Food Isolates
Source: Microorganisms. 2025 Jan 18;13(1):206. doi: 10.3390/microorganisms13010206 (PMC11768034; doi:10.3390/microorganisms13010206)
Supplement: Supplementary file 1 [file microorganisms-13-00206-s001.zip › Supplemental Information _12112024.pdf]

Supplemental Information

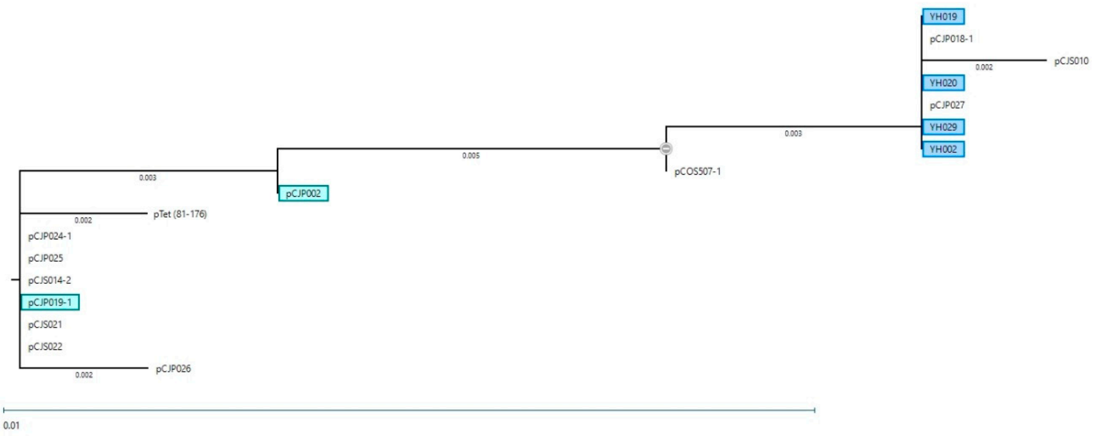

Figure S1: Phylogenetic tree of TetO protein multiple sequence alignment. Chromosomally located TetO amino acid sequences (blue outline) cluster together. For stains that had both tetO on a plasmid and the chromosome (YH002/pCJP002, YH019/pCJP019-1), the plasmid (green outline) and chromosome (blue outline) TetO amino acid sequences did not cluster together.

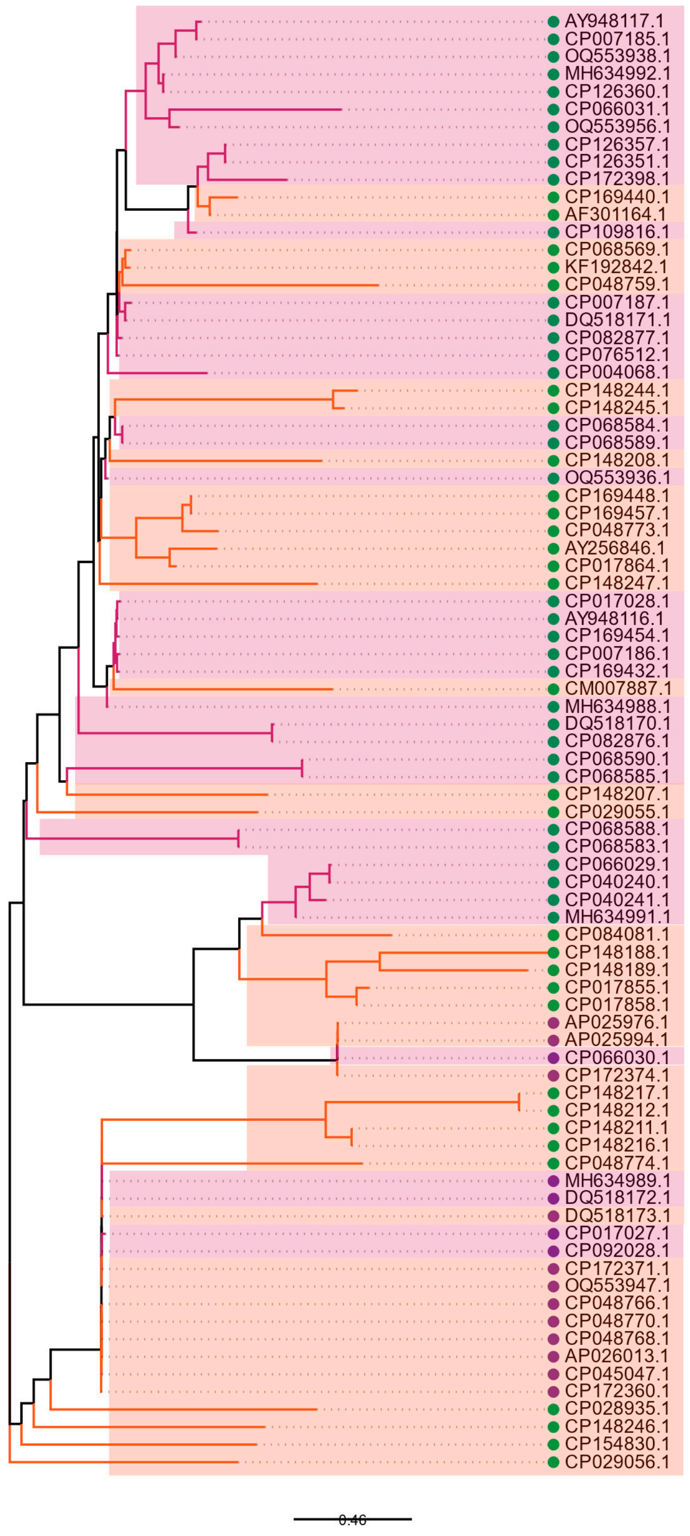

Figure S2: Phylogenetic tree of small plasmid sequences (< 6 kb) from C. jejuni and C. coli strains uploaded to NCBI. Plasmids from C. jejuni are shaded orange and plasmids from C. coli are shaded pink.
